# Endotracheal intubation with and without night vision goggles in a helicopter and emergency room setting – a manikin study

**DOI:** 10.1186/1757-7241-23-S2-A27

**Published:** 2015-09-11

**Authors:** Mikael Gellerfors, Christer Svensén, Joacim Linde, Hans Morten Lossius, Dan Gryth

**Affiliations:** 1 Karolinska Institutet/Södersjukhuset, Department of Clinical Science and Education, Section of Anaesthesiology and Intensive Care, Sweden; 2 SAE Medevac Helicopter, Armed Forces Centre for Defense Medicine (FörmedC), Sweden; 3 Ambulance Helicopter VGR, Säve, Sweden; 4 Department of Research and Development, The Norwegian Air Ambulance Foundation, Holterveien 24, PO Box 94, N-1441 Drøbak, Norway; 5 Field of Pre-Hospital Critical Care, Network of Medical Sciences, University of Stavanger, Kjell Aarholms gate 41, Stavanger, 4036, Norway; 6 Karolinska Institutet, Department of Physiology, Section of Anaesthesiology and Intensive Care, Stockholm, Sweden

## Background

Securing the airway by endotracheal intubation (ETI) is a key issue in civilian and military pre-hospital critical care. Night vision goggles (NVG) are used by personnel operating in low-light tactical environments. We examined the feasibility of an anaesthesiologist performed ETI using binocular NVG in a helicopter setting.

## Methods

Twelve anaesthesiologists performed ETI on a manikin in an emergency room (ER) setting and two helicopter-settings, with randomization to either rotary wing daylight (RW-D) or rotary wing in total darkness using binocular NVG (RW-NVG). Primary endpoint was intubation time. Secondary endpoints included success rate, Cormack-Lehane (CL) score and subjective difficulty according to the Visual Analogue Scale (VAS).

## Results

The median intubation time was shorter for the RW-D compared to the RW-NVG setting (16,5 s vs 30,0 s; p=0,03). We found no difference in median intubation time for the ER and RW-D settings (16,8 s vs 16,5 s; p=0,91). For all scenarios success rate was 100%. CL and VAS varied between the ER setting (CL 1,8, VAS 2,8), RW-D setting (CL 2,0, VAS 3,0) and RW-NVG setting (CL 3,0, VAS 6,5).

## Conclusion

This study suggests that anaesthesiologists successfully and quickly can perform ETI in a helicopter setting both in daylight and in darkness using binocular NVG, but with shorter intubation times in daylight.

## Conflicts of Interest

The authors have no conflicts of interests

